# „Die ungeklärten Gefahrenpotentiale der Gentechnologie“. Öffentliche Wissenschaft, Inszenierungsstrategien und Rhetorik der Objektivität im Kontext der bundesdeutschen Gentechnologie-Debatte

**DOI:** 10.1007/s00048-022-00346-7

**Published:** 2022-11-02

**Authors:** Anna Maria Schmidt

**Affiliations:** Universität Duisburg-Essen, Essen, Deutschland

**Keywords:** Expert*innen, Gentechnologie, Kritische Wissenschaft, Öko-Institut, Zeitgeschichte der Wissenschaften, Critical Science, Experts, Genetic Engineering, Contemporary History of Science, Oeko-Institut

## Abstract

Im März 1986 veranstalteten verschiedene ‚ökologiebewegte‘ Institutionen in Heidelberg ein öffentliches Fachsymposion mit dem Titel „Die ungeklärten Gefahrenpotentiale der Gentechnologie“, auf dem internationale Wissenschaftler*innen unterschiedlichster Disziplinen referierten. Anhand dieses Fachsymposions zeigt der Artikel, wie sich das öffentliche Auftreten von Wissenschaftler*innen als eine Form von politischem Aktivismus lesen lässt. Anhand dieser Perspektive wird herausgearbeitet, wie Biolog*innen, Chemiker*innen, Mediziner*innen, Rechts- und Politikwissenschaftler*innen politische Botschaften zu platzieren suchten, indem sie sich gerade als unabhängige Wissenschaftler*innen in Szene setzten. Das Heidelberger Fachsymposion, so die vertretene These, war darum beides: ein Ort der Wissenschaftsvermittlung und der politischen Agitation, und das in einer Zeit, da die schwarz-gelbe Bundesregierung an einer Gentechnikgesetzgebung arbeitete und auf unabhängige Expertisen angewiesen war. Der Beitrag macht deutlich, wie der Rekurs auf wissenschaftliche Unabhängigkeit zu einer Strategie in der kontroversen Debatte um den Einsatz von Gentechnik wurde. Er nimmt dabei eine Dimension politisch-wissenschaftlicher Aktivität in den Blick, die das in der Wissenschaftsgeschichte etablierte Expert*innen-Konzept nicht berücksichtigt.

## Abstract (en)

In March 1986, a public symposium took place in Heidelberg about the “unresolved potential dangers of genetic engineering”. The event was organized by institutions affiliated with the environmental movement. Choosing this symposium as an example, the article shows how the public appearance of scientists can be understood as a form of political activism. The article shows how specialists from fields as diverse as biology, chemistry, physics, law and political sciences tried to place political messages by putting themselves in the limelight as independent scientists. I argue that the symposium was both: a place of science communication intertwined with political agitation that, as should be noted, happened in a time when the West German government was working on the legislation of genetic engineering, legitimated by relying on independent expertise. It can be concluded that the discourse of scientific independence became a strategic tool in the controversial debate about the uses and dangers of genetic engineering. Thus, it draws attention to a dimension of political-scientific activity which cannot be fully grasped by the concept of ‘the expert’ that is established in the history of science.

Im März 1986 veranstalteten verschiedene ‚ökologiebewegte‘ Institutionen in Heidelberg ein öffentliches Fachsymposion mit dem Titel „Die ungeklärten Gefahrenpotentiale der Gentechnologie“. Auf dem Symposion referierten internationale Wissenschaftler*innen unterschiedlichster Fachdisziplinen zu den Gefahren der Gentechnik. Es war das erklärte Ziel der Veranstalter*innen diese auf Grundlage wissenschaftlicher Erkenntnisse öffentlichkeitswirksam zu diskutieren, um so, laut Vorwort des diesem Beitrag als Quellengrundlage zugrundeliegenden Tagungsbandes, die gesellschaftliche Debatte über die Etablierung der Gentechnik und ihrer Anwendungen in der Bundesrepublik Deutschland anzustoßen (Kollek et al. 1986b: V). Bei den für das Symposion verantwortlichen Organisator*innen – Regine Kollek, Beatrix Tappeser und Günter Altner – handelte es sich um Wissenschaftler*innen und erklärte Kritiker*innen der Gentechnologie, die sich schon in anderen Zusammenhängen öffentlich als solche präsentiert und sich somit nicht nur wissenschaftlich, sondern auch politisch gegen die kommerzielle Implementierung dieser Technologie positioniert hatten (vgl. Tappeser 1986; vgl. Hansen & Kollek 1985; vgl. Kollek 1985a: 32; vgl. Kollek 1985b).

Bei dem öffentlichen Fachsymposion handelt es sich, wie ich nun zeigen werde, um eine spezifische Form von politischem Aktivismus, die auf die Unabhängigkeit der Wissenschaften und Forschung rekurrierte und diese bewusst inszenierte. Ich analysiere, wie die beteiligten Biolog*innen, Chemiker*innen, Mediziner*innen, Rechts- und Politikwissenschaftler*innen politische Botschaften zu platzieren suchten, indem sie sich durch den Einsatz bestimmter Rhetoriken, Selbstdarstellungsweisen und Abgrenzungsstrategien gerade als unabhängige Wissenschaftler*innen in Szene setzten. Das Heidelberger Symposion erscheint, so die These, als beides: ein Ort der Wissensvermittlung und der politischen Agitation gegen die Gentechnologie, und das in Zeiten, in denen die schwarz-gelbe Bundesregierung die Möglichkeit einer gesetzlichen Regelung der Gentechnologie eruierte.^1^ Dieser als Fallstudie angelegte Beitrag möchte mit seinem Fokus auf die Rhetorik der Objektivität deutlich machen, wie der Rekurs auf Wissenschaftlichkeit und wissenschaftliche Unabhängigkeit zu einer Strategie in der kontroversen Debatte um den Einsatz von Gentechnik wurde und damit eine Dimension politisch-wissenschaftlicher Aktivität entwickelte, die sich mit dem Konzept politikberatender Expert*innen bislang nicht recht fassen lässt.^2^

In meiner Analyse des Heidelberger Symposions mache ich das Spannungsfeld zwischen Wissenschaft und Öffentlichkeit zum Gegenstand und analysiere, wie Wissenschaftler*innen über die rhetorische Inszenierung unabhängiger Wissenschaft und alltäglicher wissenschaftlicher Praxis versuchten, Deutungshoheit in politischen beziehungsweise gesellschaftlichen Fragen zu erringen. Diese Strategie interpretiere ich als Reaktion auf ein Phänomen, das gegenwartsdiagnostisch als „Wissensgesellschaft“ konzeptualisiert wird: die gestiegene Bedeutung wissenschaftlichen Wissens für politische und soziale Entscheidungsprozesse.^3^ Die zunehmende Hybridisierung von Wissenschaft und Politik bedingte sich, wie Peter Weingart nachweist, nicht nur in der Verwissenschaftlichung der Politik, beispielsweise durch die Etablierung von beratenden Expert*innengremien, sondern auch in der Politisierung der Wissenschaft (Weingart 2001). Im Rahmen der zunehmenden öffentlichen Beteiligung an wissenschaftlichen Themen wie u. a. Ökologie, Atomkraft, Gentechnologie und Psychiatrie wurde Wissen von unterschiedlichen Seiten produziert und in politischen Debatten in Stellung gebracht.^4^ Gleichzeitig wurde dieses übermäßige Wissen zunehmend rechenschaftspflichtig und stand unter erhöhten Legitimationszwängen (Weingart 2001: 15), was sich am Beispiel des Heidelberger Symposions zeigen lässt, dessen Organisator*innen mit der Überbetonung der Wissenschaftlichkeit dem gegenüber kritischen Positionen zur Gentechnologie häufig in Stellung gebrachten Vorwurf der Emotionalität zu entgehen suchten.

Dieser Artikel möchte also auch herausarbeiten, wie die an dem Heidelberger Symposion beteiligten Wissenschaftler*innen auf diese geänderten Erwartungen reagierten und wie sie selber zu dem Phänomen einer umstrittener werdenden Expertise beitrugen. Es gilt zu bedenken, wie die Wissenschaftler*innen sich selber als Mitglieder einer auch von ihnen selbst (re-)produzierten „Wissensgesellschaft“ wahrnahmen und sich in der ihnen innerhalb dieser Gegenwartsdiagnose zu Teil werdenden zentralen Rolle inszenierten und legitimierten. Der Artikel versteht sich damit als empirischer Beitrag zu einer politischen Wissensgeschichte (vgl. Espahangizi & Wulz 2020), liefert darüber hinaus aber auch einen Beitrag zur Untersuchung der Rolle *kritischer* Wissenschaftler*innen in der historisch noch wenig aufgearbeiteten Debatte um die Gentechnologie in der Bundesrepublik in den 1980er Jahren.^5^

Im ersten Teil des Beitrags verorte ich das Heidelberger Symposion in ebendieser bundesdeutschen Gentechnologie-Debatte in der Mitte der 1980er Jahre. Ich arbeite heraus, welche Ängste und Erwartungen an die Gentechnologie geknüpft und welche politischen und wissenschaftlichen Haltungen ihr gegenüber eingenommen wurden. Das Symposion nimmt hier eine besondere Rolle ein, da es die erste öffentlichkeitswirksame dezidiert *wissenschaftliche* Veranstaltung zur Gentechnologie in der Bundesrepublik war, die nicht im Dunstkreis des Bundesministeriums für Forschung und Technologie (BMFT) und den sich darum situierenden Expert*innen geplant wurde. Im zweiten Teil stelle ich die Hintergründe und Rahmenbedingungen des Heidelberger Symposions dar, das im dritten Teil hinsichtlich seiner rhetorischen Inszenierung und als Form des *scientific political activism*^6^ analysiert wird. Als Quelle für die Fallstudie steht vornehmlich der zeitnah zum Symposion veröffentlichte Tagungsband zur Verfügung. Auf Grundlage der dort enthaltenen Vorträge arbeite ich Rhetoriken, Selbstdarstellungsweisen und Abgrenzungsstrategien der beteiligten Wissenschaftler*innen heraus. Darüber hinaus ziehe ich Interviews mit Beatrix Tappeser, einer der hauptverantwortlichen Organisator*innen, sowie dem damaligen EU-Abgeordneten der Grünen Benedikt Härlin heran, welche ich zu den Hintergründen des Symposions geführt habe. Zudem dienen von den am Symposion Beteiligten verfasste oder herausgegebene Texte sowie Veröffentlichungen der veranstaltenden Organisationen als Quellengrundlage.

## Die Gentechnologie-Debatte in der Bundesrepublik Deutschland in den 1980er Jahren

Die ersten, die vor den Gefahren der Gentechnik warnten, waren die führenden Genforscher selbst. Mit der Verkündung geglückter Experimente zum Gentransfer, diskutierten die in dem Bereich führenden Wissenschaftler*innen Anfang der 1970er Jahre die unabsehbaren Folgen, die Eingriffe in die Erbinformationen von Organismen nach sich ziehen könnten und entschieden, mit ihren Bedenken hinsichtlich der Forschung mit gentechnisch verändertem Material an die Öffentlichkeit zu gehen (Singer & Söll 1973).^7^ Es ging ihnen dabei eher nachrangig um ethische Fragen gentechnischer Eingriffe. Im Mittelpunkt standen die biologischen und technischen Risiken, sprich die Gefahren unsachgemäßer Freisetzungen von im Labor erzeugter pathogener, also krankheitserregender DNA oder von Organismen, die nachhaltige Auswirkungen auf das ökologische Gleichgewicht haben könnten. Die Wissenschaftler*innen forderten deshalb öffentlichkeitswirksam zunächst einen freiwilligen Verzicht auf bestimmte, besonders riskante Experimente, bis man sich über notwendige Sicherheitsvorkehrungen verständigt hatte (Berg et al. 1974). Auf der für diese Vorgänge sinnbildlich gewordenen Konferenz im *Asilomar Conference Center* im Jahr 1975, diskutierten die 150 teilnehmenden internationalen Wissenschaftler*innen die potenziellen Gefahren gentechnologischer Forschung im Labor. Am Ende der Asilomar-Konferenz war ein Konsens über die zu erwartenden Risiken erzielt, aufgrund dessen Empfehlungen für die Weiterführung der Forschung beschlossen wurden. Zwar wollte man von Experimenten mit hochpathogenen Stoffen absehen, aber andere Laborarbeiten durften je nach kalkuliertem Risikograd unter bestimmten biologischen beziehungsweise physikalischen Sicherheitsvorkehrungen stattfinden (Berg et al. 1975). Diese Empfehlungen wurden auch die Grundlage für eine lediglich für die staatlich finanzierte Forschung verbindliche rechtliche Regulierung der Gentechnologie in den USA, auf deren Grundlage man zwar gewisse Risiken ausschloss, mit der Technologie verbundene ökonomische Hoffnungen jedoch nicht erstickte.

Das Echo der Asilomar-Konferenz erreichte Mitte der 1970er Jahre auch die Bundesrepublik Deutschland, wo eine Regelung nach Vorbild der USA implementiert wurde. Allerdings beschränkte sich die Debatte über die Gentechnologie hier auf enge Kreise aus Vertreter*innen aus Wissenschaft und Politik, die weitestgehend unter Ausschluss der Öffentlichkeit diskutierten (Salem 2013: 79–81). Bei einem der Hauptakteure auf politischer Seite handelte es sich um das Bundesministerium für Forschung und Technologie, das große, vornehmlich ökomische Hoffnungen in die Entwicklung der Gentechnologie setzte und diese als Chance zur längst überfälligen Umstrukturierung der alten chemischen Industrien begriff. Angesichts der zunehmenden Umweltproblematiken versprach die Gentechnologie eine saubere und umweltschonende Alternative zu den sichtbar dreckigen, als überholt geltenden chemischen Verfahrenstechniken zu sein. Die deutschen Chemie- und Pharmakonzerne zeigten sich jedoch recht passiv angesichts der staatlichen Bemühungen, die ‚Zukunftstechnologie‘ zu fördern, die als lukrative Möglichkeit erschien, die Bundesrepublik als wirtschaftlichen Standort zukunftsfähig zu gestalten (Barben 2007: 120–130; Dolata 1996: 129–182). Zur Entwicklung kommerziell interessanter Anwendungen forcierte das BMFT die Gründung sogenannter Genzentren, interdisziplinärer Forschungseinrichtungen, die gezielt staatliche und industrielle Forschung verbanden (vgl. Wieland 2009: 199–241).^8^ Vor dem Hintergrund der heftigen Proteste gegen die Kernenergie bemühten sich die Verantwortlichen im BMFT mithilfe von nicht-öffentlichen Expert*innenanhörungen darum, einen politischen Konsens hinsichtlich der Gentechnik zu erzielen und so der unkontrollierten Ausbreitung und Emotionalisierung der Diskussion vorzubeugen (Riesenhuber 1984: 48; Salem 2013: 79–81). Der gezielte Ausschluss der Öffentlichkeit in der Diskussion um die Gentechnologie sowie die enge Verbindung zwischen Staat und Wirtschaft in Sachen Forschung sollten von den Kritiker*innen später als Argumente gegen diese mobilisiert werden.

1984 wurde das erste gentechnisch produzierte Medikament für den deutschen Markt zugelassen, etwa zeitgleich die erste Produktionsanlage^9^ beantragt, womit auch die öffentliche Wahrnehmung der Gentechnologie zunahm. Das zeigte sich sowohl anhand der Zunahme medialer Berichterstattung, aber auch anhand der steigenden Anzahl von Diskussionsgruppen und Veranstaltungen, die sich diesem Thema widmeten (vgl. Salem 2013: 85–140; exemplarisch Schmidt 2020). Neben den ökonomischen und ökologischen Vorteilen der Gentechnologie verwiesen ihre Befürworter*innen auch auf deren medizinischen Nutzen, wobei die Verhinderung von Erbkrankheiten und die Herstellung passgenauer Medikamente zur Heilung von Zivilisations- und Infektionskrankheiten im Vordergrund standen. Im Gegensatz dazu warnten die Kritiker*innen vor den unkalkulierbaren Folgen unsachgemäßer Freisetzungen für das Ökosystem und die Gesundheit, dem militärischen Einsatz der Gentechnologie sowie vor den Auswirkungen genmanipulativer Eingriffe ins menschliche Erbgut (vgl. Gill 2008). Gegenstand und Grenzen der Gentechnologie waren in der öffentlichen Debatte keineswegs genau geklärt. Verwechslungen waren insbesondere mit der Biotechnologie häufig, wobei es sich bei der Gentechnik im strengen Sinne nur um *eine* biotechnologische Methode handelt. Beeinflusst wurde die Debatte über die Gentechnologie auch von den Fortschritten im Bereich der Reproduktionsmedizin, durch die gentechnische Anwendungen am Menschen und damit verbundene ethische Bedenken in den Fokus gerückt wurden. Vor dem Hintergrund der spezifisch deutschen Vergangenheit der medizinischen Verbrechen und der eugenischen Selektion im Nationalsozialismus verband insbesondere die deutschsprachige Frauenbewegung die Kritik an den Fortpflanzungstechniken mit der an der Gentechnologie, unter die so nun auch biologische Techniken subsumiert wurden, die Eingriffe ins Erbgut eigentlich nicht vorsahen, aber die zunehmende Kontrolle der Fortpflanzung versinnbildlichten (vgl. Brandt 2010). Der Begriff Gentechnologie avancierte in den 1980er Jahren vielmehr zur Chiffre für die Technisierung des Lebendigen im Allgemeinen (Vowe 1989: 50).

Zur Ausweitung der Debatte auf größere gesellschaftliche Zusammenhänge trug auch die Partei Die Grünen bei, die sich das Thema rasch nach ihrem Einzug in den Bundestag im Jahr 1983 auf die politische Fahne schrieb und als Oppositionspartei auf die Mobilisierung gesellschaftlicher Unterstützung angewiesen war, um ihre eigene Position gegenüber den anderen Parteien zu stärken (vgl. Steindor 1999). Die Grünen, allen voran die Abgeordnete Erika Hickel, Apothekerin und damals Professorin für Geschichte der Pharmazie und der Naturwissenschaften an der TU Braunschweig, erkannten im industriellen Gebrauch der Gentechnologie bereits deren Missbrauch (Hickel 1985: 341). Sie forderten einen sofortigen Stopp der nicht ausreichend durch gesellschaftliche Mitsprache legitimierten staatlich finanzierten Förderung der Gentechnik und ihrer industriellen Anwendung. Dahinter stand die Forderung, Technikentwicklungen generell zum Gegenstand gesamtgesellschaftlicher Partizipation zu machen (Steindor 1999: 369). Um ihren Interessen Gewicht zu verleihen, richtete die Partei einen wissenschaftlichen Stab ein, in dem auch Beatrix Tappeser, eine der Organisator*innen des Heidelberger Symposions arbeitete. Dieser auch als Bundesarbeitsgemeinschaft „Gen- und Reproduktionstechnologien“ bekannte parteieigene *Thinktank* erarbeitete Positionen und Strategien gegen die Durchsetzung der Gentechnologie und koordinierte die parlamentarischen und außerparlamentarischen Initiativen der Partei in diesem Bereich – immer unter der Maßgabe möglichst großer Öffentlichkeitswirksamkeit (Steindor 1999: 369).

Um die Debatte in die Öffentlichkeit zu tragen, beantragten SPD und Grüne 1984 die Einsetzung einer Enquete-Kommission.^10^ Die Aufgabe der noch im gleichen Jahr eingesetzten Kommission „Chancen und Risiken der Gentechnologie“ – die Namensgebung folgte dem SPD-Antrag und widersprach den Grünen, die die Gentechnologie nicht im Spannungsfeld möglicher Chancen und Risiken betrachten wollten, sondern sie in allen industriellen Anwendungsgebieten ablehnten – bestand in der Untersuchung der ökonomischen, ökologischen, rechtlichen und gesellschaftlichen Auswirkungen sowie der Sicherheitsgesichtspunkte und ethischen Aspekte der gen- und biotechnologischen Forschung (Deutscher Bundestag 1987: III–V). Sie erschloss bis Ende des Jahres 1986 grundrechtliche Zielkonflikte, bestimmte Grenzen für den Einsatz beim Menschen sowie Prioritäten zur Förderung sinnvoller Anwendungen, entwickelte Vorschläge für Sicherheitsstandards und erarbeitete Empfehlungen für politische Entscheidungen (Deutscher Bundestag 1987), die dann in das 1990 verabschiedete Gentechnikgesetz einflossen.^11^ Die neun Fraktionsmitglieder und acht sachverständigen Kommissionsmitglieder aus unter anderem Biologie, Medizin und Rechtswissenschaften wurden unterstützt von einem wissenschaftlichen Sekretariat, das auf Vorschlag der Fraktionen berufen wurde. Dort arbeitete die Molekularbiologin Regine Kollek, die gemeinsam mit Günter Altner und Beatrix Tappeser das Heidelberger Symposion organisierte. Die Idee zur Veranstaltung des Symposions entstand in diesen gerade beschriebenen Zusammenhängen zwischen der Bundesarbeitsgemeinschaft der Grünen und der Enquete-Kommission.^12^

## Hintergründe und Rahmenbedingungen des Heidelberger Fachsymposions

Bei den drei Veranstalter*innen des Fachsymposions handelte es sich allesamt um promovierte Biolog*innen. Dies gilt auch für Günter Altner, der darüber hinaus in der Evangelischen Theologie promoviert war und aufgrund seines Lehrstuhls in dem Bereich häufig als Theologe verortet wird. Altner beschäftigte sich seit seinem Studium der Biologie und Evangelischen Theologie mit Grenzfragen zwischen Wissenschaft und Religion und engagierte sich für den Umweltschutz und in der Anti-Atomkraft-Bewegung. Seit 1977 war er Vorstandsmitglied des von ihm mitgegründeten Öko-Instituts Freiburg (Institut für angewandte Ökologie e. V.), das als Geldgeber des Symposions fungierte. Zwischen 1979 und 1983 war er als Interessenvertreter des Widerstands gegen die Atomtechnologie Teil des Sachverständigengremiums der Enquete-Kommission „Zukünftige Kernenergie-Politik“, deren Arbeit – gerade von Kritiker*innen der Kommission zur Gentechnologie – häufig als beispielhaft herausgestellt wird, weil sie Vorschläge für Alternativen zur Kernenergie aufzeigte (vgl. Vowe 1991: 191–438).

Regine Kollek hatte neben Biologie auch Chemie studiert. Anschließend forschte sie zwischen 1979 und 1981 an der Medical School der University of California in San Diego, dann am Heinrich-Pette-Institut für Experimentelle Virologie in Hamburg über die Mechanismen der Krebsentstehung.^13^ Kollek verließ das Institut 1984, weil sie nicht zu Forschungszwecken an der künstlichen Herstellung für den Menschen pathogener Stoffe beteiligt sein wollte, für die immer das Risiko einer unabsichtlichen Freisetzung bestand (Maurer 1986: 10). Nach ihrer Mitwirkung im wissenschaftlichen Stab der Enquete-Kommission arbeitete sie in der Technikfolgenabschätzung und bis 1988 als freiberufliche Mitarbeiterin am Öko-Institut.

Beatrix Tappeser hatte nach ihrem Studium im Bereich der Immunologie promoviert. Von 1982 bis 1984 war sie als wissenschaftliche Mitarbeiterin am Institut für Molekularbiologie und Biochemie in der medizinischen Grundlagenforschung an der FU Berlin tätig. In dieser Zeit entwickelte sich auch ihre kritische Haltung gegenüber der Gentechnologie.^14^ Sie beteiligte sich in einer im universitären Kontext angesiedelten Frauengruppe zum Thema Gen- und Reproduktionstechnologien, zu der sie wegen ihres wissenschaftlichen Hintergrunds und ihrer kritischen Haltung gegenüber der eigenen Fachdisziplin eingeladen wurde. Als Frau in einem männerdominierten Wissenschaftssystem sympathisierte sie mit der Frauenbewegung.^15^ Die Chance auf eine Habilitation schlug sie schließlich aus und verließ die Universität, weil sie sich, wie sie selbst betont, mit dem Wissenschaftssystem im Allgemeinen und besonders mit dem die Molekularbiologie beherrschenden atomisierenden Ansatz bausteinartiger Zerteilung und Rekombination von DNA nicht mehr arrangieren wollte. Sie war, wie Regine Kollek, Vertreterin eines kontextorientierten Ansatzes, der sich für die je nach Umgebung unterschiedliche Funktion von genetischen Informationen interessierte.^16^ Ihr Weg führte Tappeser in die Bundesarbeitsgemeinschaft der Grünen, in der sie als wissenschaftliche Mitarbeiterin beschäftigt war. Nebenbei engagierte sie sich seit 1985 als ehrenamtliches Mitglied im Vorstand des Öko-Instituts, wo sie ab 1987 dann auch einen mit der Gentechnologie befassten Forschungsbereich leitete.

Sowohl Kollek und Tappeser als auch Altner sprachen sich nicht generell gegen die Gentechnologie als Methode aus, sondern erkannten in ihr eine wichtige analytische Methode der Grundlagenforschung. Allerdings warnten sie vor dem Risiko unsachgemäßer Freisetzungen in offene Systeme. Eine neue Dimension sahen sie in der industriellen Anwendung der Gentechnologie erreicht. Als problematisch erachteten sie insbesondere die gemeinsam von Staat und Unternehmen getragene Finanzierung der Forschung, durch welche weniger die Erforschung der Grundlagen als vielmehr die Entwicklung konkreter Anwendungen und Produkte in den Fokus gerückt würde (Maurer 1986: 8).

In ihrer Rolle als Wissenschaftler*innen wollten Regine Kollek, Beatrix Tappeser und Günter Altner mit ihrem Symposion eine neue Öffentlichkeit für „kritische“ und gleichzeitig „wissenschaftliche“ Positionen schaffen. Tatsächlich tauchte der im Sommer nach dem Symposion herausgegebene Tagungsband nicht nur in den Lektürevorschlägen der Gentechnik-kritischen Szene auf (Anonym 1986: 1), sondern wurde – nach Angaben der Organisator*innen – auch medial breit rezipiert.^17^ Mit ihrem Symposion reagierten die Veranstalter*innen auf die ihrer Meinung nach einseitige Berichterstattung über Wissenschaftler*innen, die die Gentechnik häufig nur vor dem Hintergrund vermeintlicher Nutzungsziele und Anwendungsfolgen betrachteten. „Das spricht nicht für die Unabhängigkeit des wissenschaftlichen Sachverstands!“, formulierte Günter Altner in seinen einleitenden Worten (Altner 1986: 3). Zudem seien die Forscher*innen zumeist in den Strukturen des staatlich und industriell finanzierten Wissenschaftssystems verhaftet und könnten schon aus individuell-ökonomischen Gründen keine öffentlichen Bedenken äußern. Es war den Veranstalter*innen daran gelegen, mit dem Symposion zu zeigen, dass eine kritische Debatte unter Wissenschaftler*innen bereits bestand, um auch andere zu motivieren, sich über die sich im Rahmen der Forschung ergebenden Probleme auszutauschen (Kollek et al. 1986a: 232).^18^ Nach Angaben der Organisator*innen reagierte das Symposion zudem auf das gesellschaftliche Informationsbedürfnis nach einer von Nutzungsinteressen unabhängigen Bewertung der Gentechnologie sowie nach verbindlichen Aussagen über deren bereits real existierende Möglichkeiten. Wieland hat bereits herausgestellt, dass die Debatte der 1980er Jahre ihrer Zeit weit vorauslief. Im Mittelpunkt standen hier mehr die in ferner Zukunft liegenden Visionen als die sich immer mehr konkretisierenden Optionen (Wieland 2012: 80). Zum Beispiel kursierten erste Falschmeldungen über geklonte Mäuse (vgl. Illmensee & Hoppe 1981), die die deutschen Printmedien zum Anlass nahmen, die prinzipielle Realisierbarkeit dieser Anwendungen auch am Menschen zu antizipieren und zum Beispiel von „der durch Klonen verhundertfachten Brigitte Bardot oder dem dutzendfach kopierten Albert Einstein“ (Anonym 1981: 154) zu träumen beziehungsweise diese als Schreckensszenario heraufzubeschwören (vgl. Brandt 2010). Die Abkopplung der Debatte von den realen Möglichkeiten verstärkte deren emotionale Aufladung. Um sich mit der Kritik an der Gentechnologie wirksam Gehör zu verschaffen, mussten die Befürchtungen und Ängste in rationale Argumente überführt werden. Nach Altners Einführungsvortrag sei es leicht, „die vom Bürger geäußerten Befürchtungen […] als emotionale Einrede schnell abzutun“, weshalb konkrete Erkenntnisse zur Stützung der Argumente herangezogen werden müssten (Altner 1986: 1). Auch deshalb veranstalteten Tappeser, Kollek und Altner die Tagung mit einem explizit fachwissenschaftlichen Anspruch und grenzten sich so von anderen, eher auf Laien ausgerichteten und emotionale Argumente zulassenden Foren ab, um auch der staatlichen Politik und dem institutionalisierten Wissenschaftssystem wirksam die Stirn bieten zu können.

Einen positiven sowie negativen Bezugspunkt bildete für die Organisator*innen auch die bereits erwähnte Asilomar-Konferenz. Sie stellte gewissermaßen die Blaupause für das dem Symposion zugrundeliegende Bild kritischer Wissenschaftler*innen dar, die zur Aufklärung der Bevölkerung zusammenkamen und ihre Forschung nur unter der Bedingung eines gesellschaftlich getragenen Konsens fortführen wollten. Gleichzeitig waren sich die Veranstalter*innen des Heidelberger Symposions darin einig, dass diese mehr als zehn Jahre zurückliegende Konferenz schon zu oft als Argument für das Verantwortungsbewusstsein und die gelungene Selbstregulierung der Wissenschaften hergehalten hatte und es an der Zeit war, weitere kritische Auseinandersetzungen mit dem Thema seitens der Wissenschaftler*innen anzustoßen. Zudem zeigte sich inzwischen, dass die Asilomar-Konferenz ganz beträchtlich dazu beigetragen hatte, die amerikanische Debatte auf bestimmte Themen sowie Personenkreise zu beschränken und so einer öffentlichen Auseinandersetzung mit der Gentechnologie eher hinderlich gewesen war.^19^

Finanziell wurde das Symposium unterstützt durch das Öko-Institut Freiburg, den Bund für Umwelt und Naturschutz Deutschland e. V. (BUND), die Altner Stiftung, die Deutsche Umweltstiftung und die Stiftung Mittlere Technologie. Alle diese Institutionen waren im Zusammenhang mit der Ökologiebewegung entstanden, widmeten sich Umweltschutz-Themen und waren an der Schnittstelle zwischen Wissenschaft, Politik und Öffentlichkeit angesiedelt. Das Öko-Institut Freiburg, in dem sich alle drei Organisator*innen engagierten beziehungsweise beschäftigt waren, ist ein im Kontext der Anti-Atomkraft-Bewegung von Rechts- und Volkswissenschaftler*innen, kritischen Naturwissenschaftler*innen, Mitgliedern der Umweltbewegung und der evangelischen Kirche ins Leben gerufenes unabhängiges Forschungsinstitut, das sich bis heute der Förderung des Umweltschutzes und der Nachhaltigkeit widmet (Öko-Institut 2017: 1–2). Die Idee hinter der Gründung des Instituts war die Etablierung einer kritischen beziehungsweise alternativen Wissenschaft, die unabhängig von staatlichen oder wirtschaftlichen Geldgebern zu Umweltproblemen forschen konnte und deshalb in ihren Forschungsergebnissen auch unabhängig war (Roose 2002: 17; vgl. Grießhammer 2001).^20^ Mit der Gründung des Öko-Instituts reagierte man auf ein in der Auseinandersetzung um die Kernenergie zutage tretendes Problem. Die Risikobewertung sowie die Kontrolle oblagen hier jenen Forscher*innen, deren Arbeitgeber*innen die Technik im Vorfeld finanziert und denen man somit ein unmittelbares ökonomisches Interesse an deren Weiterführung sowie eine berufliche und finanzielle Abhängigkeit unterstellte. Für die Gründer*innen des Öko-Instituts konnte eine sich so sehr in Abhängigkeiten befindliche Forschung keine unabhängigen Studien liefern. Das Öko-Institut stellte seit seiner Gründung eine wichtige wissenschaftliche Ressource für die sozialen Bewegungen, und später auch darüber hinaus dar. Seine Hauptaufgaben liegen in der Erarbeitung von unabhängigen Gutachten und der Vermittlung von Sachverständigen zu ökologisch relevanten Fragen (Roose 2002: 68). Sein Engagement in Sachen Gentechnologie nahm die Einrichtung 1986 mit der Unterstützung des Symposions auf. Etwa zeitgleich erarbeitete das Öko-Institut, das sich zu dieser Zeit in den Debatten um die Sicherstellung der Energieversorgung schon öffentlich hervorgetan hatte (Kampe 1986: 34), im Auftrag des Hessischen Ministers für Umwelt und Energie, des Grünen-Politikers Joschka Fischer, ein Gutachten über die Gefahren der Gentechnik (Öko-Institut 2017: 4).

Der BUND war bereits 1975 von Mitgliedern der Wissenschaften, der Umweltbewegung und Wissenschaftsjournalisten gegründet worden und diente der kritischen Beobachtung umweltpolitischer Entwicklungen sowie der öffentlichen Information in Sachen Umwelt- und Naturschutz. Auch die an dem Symposion beteiligten Stiftungen engagierten sich für Umweltschutz, ökologisches Gleichgewicht oder den Einsatz natur- und menschengerechter Technologien in der Landwirtschaft.^21^ Die am Symposion beteiligten Organisator*innen und Institutionen stellten, indem sie sich von politischen Institutionen wie Industrie unabhängig erklärten, eine Form politisch engagierter Wissenschaft dar, die in einem besonderen Maße auf Öffentlichkeit angewiesen war, um die eigene politische Relevanz und Deutungshoheit zu etablieren (vgl. Rucht 1988: 298–300; vgl. Güttler: Special Section Gegenwissen).

Die Planung des Symposions erfolgte zu großen Teilen in Tappesers Wohnzimmer in Bonn, da sie im Zeitraum der Vorbereitungen ein Kind bekam.^22^ Dort erarbeiteten die drei Organisator*innen thematische Zuschnitte für die einzelnen Panels und erstellten auf dieser Grundlage eine Liste mit dafür in Frage kommenden Referent*innen. Ein Großteil der Angeschriebenen nahm die Einladung an; gemäß Tappeser ergaben sich Absagen nur aus terminlichen Gründen, nicht aus Vorbehalten gegen das Format.^23^ Das räumliche Setting arrangierten die Veranstalter*innen im klassischen Vortragsformat. Es gab ein Podium, auf dem die Referent*innen standen beziehungsweise die Teilnehmer*innen an den Podiumsdiskussionen saßen, davor der Zuschauerraum, in dem sich das Publikum sitzend versammelte.^24^

Aufgrund der Arbeitsschwerpunkte der veranstaltenden Organisationen und der fachwissenschaftlichen Kompetenz der Organisator*innen begründete sich auch der thematische Zuschnitt des Symposions, das auf Gefahren im mikrobiellen Bereich sowie die Grüne Gentechnik, also deren Einsatz in der Pflanzen- und Tierzucht fokussierte. Gentechnologische Einsätze beim Menschen wurden laut Einführungsvortrag „so prüfenswert dieser Anwendungsbereich auch ist“ aus Zeitgründen nicht behandelt (Altner 1986: 2).

Der Anspruch des Symposions war, die möglichen Gefahren der Gentechnologie „umfassend“ zu betrachten, weshalb nicht nur die ökologischen Folgen, sondern auch die sozialen und rechtlichen Auswirkungen der Technologie in den Blick genommen wurden. Diese Schwerpunktsetzung manifestierte sich auch im Tagungsprogramm, das neben dem Einführungsvortrag aus dreizehn Vorträgen bestand, die sich aus historischer oder sozialwissenschaftlicher Perspektive kritisch mit der Gentechnologie auseinandersetzten, verschiedene biologische, medizinische und ökologische Bedrohungsszenarien in den Blick nahmen oder Möglichkeiten rechtlicher und politischer Rahmung der Technologie diskutierten.^25^ Neben den Vorträgen bildeten die Diskussionen im Plenum, die im Tagungsband leider nicht vollständig wiedergegeben sind (Kollek et al. 1986b: V), einen wichtigen Teil des Tagungsprogramms.^26^ Zum Tagungsprogramm gehörten auch zwei Gespräche auf dem Podium: ein Streitgespräch zur Entstehungsgeschichte von AIDS und eine Diskussion über die Notwendigkeit, Maßstäbe und Grenzen einer umfassenden Risikoabschätzung in der Gentechnologie.

Unter den Referent*innen fanden sich international renommierte Biowissenschaftler*innen, wie beispielsweise der Londoner Biologieprofessor Steve Rose, der japanische, seit 1966 in Australien wirkende Zoologe Atuhiro Sibatani und die australische Molekularbiologin und Wissenschaftshistorikerin Ditta Bartels. Daneben sprachen von deutscher Seite unter anderem das Ehepaar Christine und Ernst-Ulrich von Weizsäcker, das eine Bewertung der Gentechnologie aus evolutionsbiologischer Sicht vornahm. Weizsäcker war nicht nur Neffe des damaligen Bundespräsidenten Richard von Weizsäcker, sondern auch von 1984 bis 1991 Direktor des Instituts für Europäische Umweltpolitik. Zudem referierte die Molekulargenetikerin und Sachverständige in der Enquete-Kommission „Chancen und Risiken der Gentechnologie“, Gisela Nass-Hennig, der SPD-Politiker und anfängliche Vorsitzende der Enquete-Kommission „Zukünftige Kernenergie-Politik“, Reinhard Ueberhorst sowie der Rechtwissenschaftler Gerd Winter, der sich mit rechtlichen Fragen der Gentechnologie auseinandersetzte. Neben den anwesenden Referent*innen beteiligten sich als Diskutant*innen zum Beispiel der Grünen-Abgeordnete und Biologe Arnim von Gleich, der Wissenschaftsjournalist Ruben Scheller und der Biologe, Genetiker und Wissenschaftssoziologe Rainer Hohlfeld, der wie Regine Kollek im Sekretariat der Enquete-Kommission zur Gentechnologie arbeitete. An den Diskussionen beteiligte sich auch Erika Hickel, die als Grünen-Abgeordnete bis zu ihrem, durch das Rotationsprinzip bedingten Ausscheiden ebenfalls der Enquete-Kommission zur Gentechnik angehört hatte, sowie Heidemarie Dann, ihre Nachfolgerin im Bundestag und in der Kommission. Die Anwesenden verband ihr grundlegendes Interesse an den Entwicklungen der Gentechnologie. Fast alle hatten einen wissenschaftlichen Hintergrund, auch wenn sich einige eindeutiger im akademischen Umfeld, andere eher innerhalb der Politik verorteten.

Da es den Veranstalter*innen bei dem Symposion stark um die Außenwirkung ging, waren auch Vertreter*innen der Presse geladen, die über das Tagungsgeschehen berichteten.^27^ Darunter die Herausgeber*innen des Gen-ethischen Informationsdienstes^28^ (Anonym 1986), die zudem die Diskussionen für den Tagungsband protokollierten (Kollek et al. 1986b: V). Hier zeigt sich der Wunsch nach einer möglichst breiten gesellschaftlichen, außer-wissenschaftlichen Rezeption des Symposions sowie eine gewisse Vernetzung mit der Gentechnik-kritischen Szene. Der öffentlichen Information diente auch der in der Reihe „Gentechnologie“ vom J. Schweitzer Verlag München veröffentlichte Tagungsband, der die Vorträge und Standpunkte der Diskussionen wiedergab. In der gleichen Reihe wurden auch die Ergebnisse der internen Veranstaltungen des BMFT veröffentlicht. Auf der Grundlage des Tagungsbandes werde ich im nächsten Abschnitt herausarbeiten, wie die Organisator*innen mit der Veranstaltung politisch agierten, welche politischen Ziele sie verfolgten und mit welchen Inszenierungsstrategien sie diese umzusetzen versuchten.

## Das Heidelberger Symposion als Inszenierung öffentlicher Wissenschaft

Wissenschaftliche Tagungen, Konferenzen, Kongresse oder auch Symposien dienen im Allgemeinen dem fachlichen Austausch von Forscher*innen, die zu einem gemeinsamen Thema arbeiten oder einer speziellen Frage- beziehungsweise Problemstellung nachgehen. Wohingegen geschlossene Konferenzen auf eine schnelle, effektive und innovative Wissensproduktion ausgelegt sind, lassen sich Formate mit Publikum wie das Heidelberger Symposion als Weiterentwicklung in Richtung öffentliche Wissenschaft verstehen (Mead & Byers 1968: 3–8).

Das zeigt sich schon an der Asilomar-Konferenz, über die die zuvor nur in geschlossenen wissenschaftlichen Kreisen geführten Diskussionen in die Öffentlichkeit getragen werden sollten. Das Vorbild griffen die Veranstalter*innen des Heidelberger Symposions auf und rekurrierten auf diese öffentliche Art des Konferenzgeschehens, wobei – wie ich gleich zeigen werde – die öffentliche Meinungsbildung mehr im Fokus stand. Wie bei den amerikanischen Wissenschaftler*innen war es auch das Anliegen der Veranstalter*innen des Fachsymposions, politisch wirksam über die Gefahren der Gentechnologie aufzuklären, auch aufgrund des zunehmenden Zeitdrucks in Bezug auf die „drohenden“ staatlichen Regelungen der Gentechnologie.

Indem das Heidelberger Symposion explizit als öffentlich angelegt war, grenzte es sich zudem von den zuvor durch das BMFT veranstalteten nicht-öffentlichen Expert*innenanhörungen ab. „Wir praktizieren hier ein Stück öffentlicher Wissenschaft“ formulierte Günter Altner in seinem Einführungsvortrag in das Fachsymposion, wobei der Verweis auf die Öffentlichkeit die Veranstaltung im demokratischen Sinne legitimierter erscheinen lässt als die in dieser Kontrastierung als „Geheimabkommen“ erscheinenden Treffen zwischen Politik, Wirtschaft und Wissenschaft, die sich einzig an Nutzungsinteressen orientierten (Altner 1986: 1).

Das Heidelberger Symposion erscheint aus dieser Perspektive nicht nur als Vollzug einer routinierten wissenschaftlichen Praxis (vgl. De Boer 2019: 28–34), sprich einer Konferenz, sondern auch als Ort der öffentlichen Inszenierung von Wissenschaft und Objektivität zur Erlangung von Deutungsmacht in wissenschaftspolitischen Fragen.

Die am Symposion Beteiligten verfolgten unterschiedliche politische Ziele: Manche Teilnehmer*innen wollten ein generelles Verbot der Gentechnologie sowie einen Stopp der staatlich finanzierten Forschung erreichen, andere setzten sich für die Beschränkung ihrer industriellen Anwendung ein und/oder sprachen sich für umfassende Risikoanalysen in Bezug auf mögliche Freisetzungen aus. Über wissenschaftliche Argumente und deren Verortung in einer explizit wissenschaftlichen Praxis – einem Fachsymposion – versuchten sie, ihr Wissen als unabhängig von politischen und wirtschaftlichen Interessen dazustellen. Über die Inszenierung einer von Nutzungsinteressen unabhängigen Wissenschaft adressierten die Veranstalter*innen ihre politischen Botschaften an eine Öffentlichkeit, deren Misstrauen so erst fundiert wird.

Das Heidelberger Symposion diente zunächst der Bildungsarbeit zum Thema Gentechnologie. Es war so konzipiert, dass die Zuschauer*innen, genauso wie die Rezipient*innen der publizierten Tagungsberichte, allein durch das Verfolgen des Konferenzgeschehens, beziehungsweise die spätere Lektüre des Tagungsbandes, einen fundierten Einblick in die wissenschaftliche Debatte über die Gentechnik erhalten und über die „kontrovers-kritische Erörterung“ (Altner 1986: 1) in ihrer Entscheidungsfindung unterstützt werden sollten. Besonders gut kommt dies im Einführungsvortrag Günter Altners zum Ausdruck, der „die Bürger, die Abgeordneten in den Parlamenten und die Mitglieder der Regierungen“ als gewünschtes Publikum nannte (Altner 1986: 1). Aus Sicht der Veranstalter*innen waren diese auf von Nutzungsinteressen unabhängiges Wissen angewiesen, das die als mit der Wirtschaft verstrickt geltenden Expert*innen eben aufgrund ihrer Abhängigkeit nicht in der Lage waren zur Verfügung zu stellen.

Die Abgrenzung von der kommerziellen Forschung zeigte sich innerhalb der Vorträge auf dem Heidelberger Symposion als zentrales Motiv der Begründung der eigenen Unabhängigkeit. Die nach kurzfristigen wirtschaftlichen Zielen operierende Industrieproduktion wurde der eigenen – ökologische Zusammenhänge bedenkenden – Forschungsweise gegenübergestellt, wie sich exemplarisch am Beitrag der beiden Veterinärmediziner*innen Anita Idel und Rolf Kamphausen mit dem bezeichnenden Titel „Angepasste Landrassen – oder Nutztiere aus der Retorte?“ veranschaulichen lässt, der den „fragwürdigen Beitrag der Gentechnologie zur Tierzüchtung“ (Idel & Kamphausen 1986) untersuchte. Die Redner*innen stellten heraus, dass die Berücksichtigung rein wirtschaftlicher Ziele in der Hochleistungszucht nicht nur zur Reduktion der genetischen Basis, sondern auch zum Verlust des Blicks auf die Bedeutung dieser Rassen für die Ökosysteme führe, was für die Zuchtbetriebe ebenso wenig eine Rolle spiele, wie die Tiere in ihrer Komplexität. Dem setzten Idel und Kamphausen entgegen: „Milch wird nicht durch ein Gen oder ein Organ, sondern durch die ganze Kuh gebildet“ (Idel & Kamphausen 1986: 149). Ebenso wie die Veranstalter*innen des Symposions lehnten Idel und Kamphausen einen biologistischen Ansatz ab, der die Möglichkeit einer baukastenartigen Zerteilung und Rekombination von Erbmaterial suggeriert, und kritisierten die nicht ganzheitliche Betrachtungsweise der rein auf Wirtschaftlichkeit ausgelegten industriellen Landwirtschaft und Tierzucht, in der das „Gesamttier […] nur unter dem Punkt Sentimentales auf[taucht]“ (Idel & Kamphausen 1986: 148).

Trotz des nach außen artikulierten ostentativ wissenschaftlichen und bildungspolitischen Ansatzes lässt sich – und das macht bereits der von den eben in den Fokus gerückten Referent*innen gewählte Titel deutlich – nicht leugnen, dass die Betrachtung der Gefahrenpotenziale eine negative Haltung zur Gentechnologie nahelegte. Die vorgetragenen Themen waren so gewählt, dass sie alarmierend wirkten: Angefangen bei dem Abendvortrag zu „Gentechnologie und biologische Waffen“ (Rose 1986) über das staatlich-ökonomische Interessenkartell in Sachen Gentechnik (Wright 1986) bis hin zur Darstellung einer quasi natürlichen Verbindung gentechnologischer Forschung mit Krankheiten wie Krebs und AIDS (Bartels 1986; Kollek 1986b; Kollek 1986a: 89–94).^29^ Letztere waren auch für Laien überaus anschlussfähige Stichworte. Die unheimliche Bedrohung durch den AIDS-Erreger hatte seit Beginn der 1980er Jahre weltweite Ängste ausgelöst und vor der Folie gentechnologischer Forschung und dem Kalten Krieg entsponnen sich verschiedene teilweise politisch motivierte Gerüchte über die Entstehung des HI-Virus in amerikanischen Forschungslaboren (vgl. Geißler 2014; vgl. Selvage & Nehring 2014; vgl. Beljan 2015). Das Streitgespräch zwischen John Collins, dem Leiter des Forschungsbereichs Genetik und Zellbiologie am Helmholtz-Zentrum für Infektionsforschung in Braunschweig und Regine Kollek knüpfte an diese Meldungen an und erörterte die Plausibilität der einzelnen Argumente. Auch wenn Regine Kollek keinesfalls behauptete, dass der HI-Virus *in vitro* entstanden sei, so demonstriert für sie die Debatte darüber, dass die Existenz mittels Gentechnologie hergestellter Viren nicht auszuschließen sei und die Manipulation letztendlich auch nicht nachweisbar wäre (Kollek et al. 1986a: 94).^30^ Obgleich Collins die Theorie einer künstlichen Herkunft der Krankheit in der auf dem Symposion stattfindenden Diskussion als nicht sehr plausibel bewertete, hinterließ Kollek das Publikum mit dem Horrorszenario von im Labor erzeugten und willkürlich freigesetzten Krankheiten. Besondere Brisanz erhält die Darstellung durch ein Spezifikum retroviraler Erkrankungen wie zum Beispiel AIDS, das Kollek in ihrem Vortrag erwähnte (Kollek 1986b: 49): deren mehrjähriger Latenzzeit bis zum symptomatischen Ausbrechen der Krankheit. Ein durch ein Retrovirus infizierter Organismus könnte also eine Vielzahl weiterer Ansteckungen provozieren, ohne dass dies überhaupt bemerkt werden würde (Kollek 1986: 49). Über diese Darstellung evozierte Kollek Handlungsdruck in Bezug auf die Regulierung gentechnologischer Forschung, die aufgrund der Tatsache, dass das Szenario unbemerkt längst hätte eingetreten sein können, von oberster Dringlichkeit schien (Schmidt 2019). Hier verbinden sich wissenschaftliche Argumentation und politische Agitation. Das Publikum sollte mithilfe wissenschaftlicher Beweisführung zu einer kritischen Haltung zur Gentechnologie bewogen werden, sodass über die Mobilisierung einer gesellschaftlichen „Gegenmacht“^31^ der Handlungsdruck auf die Politik erhöht würde, nicht aufgrund ökonomischer Interessen über die mit der Technologie einhergehenden Risiken hinwegzugehen und leichtfertig über eine Gentechnologiegesetzgebung zu entscheiden.

Während sich Befürworter*innen der Gentechnologie dem Vorwurf aussetzten, deren mögliche Gefahren zugunsten ökonomischer Chancen zu vernachlässigen, sahen sich Kritiker*innen mit der Anschuldigung übertriebener Emotionalität und Unwissenschaftlichkeit konfrontiert, wie Günter Altner in seiner Einführungsrede schon deutlich machte (Altner 1986: 1). Beim Fachsymposion ging es deshalb auch darum, dieses explizit als wissenschaftliche Veranstaltung zu inszenieren, auch um dem antizipierten Vorwurf der Emotionalität entgegenzuwirken. Deshalb wurde schon in der Einführungsrede darauf hingewiesen, dass es sich bei den Vortragenden nicht etwa um Laien handle, sondern um in ihren Disziplinen langjährig akademisch ausgebildete Wissenschaftler*innen, wodurch die Statuszugehörigkeit der Referent*innen explizit betont wurde. Auch die vorgestellten Erkenntnisse wurden deshalb im Rahmen einer ausdrücklich wissenschaftlichen Praxis, eben des Fachsymposions, verortet, um die eigene wissenschaftliche Autorität gegenüber der Fach-Community, der Bevölkerung und den angesprochenen politischen Instanzen klarzustellen. Dennoch handelte es sich nicht um eine wissenschaftliche Konferenz im klassischen Sinne (Mead & Byers 1968 3–6), von der sich das Heidelberger Symposion gerade aufgrund seiner öffentlichen Ausrichtung unterschied.

Um politisch wirksam zu sein, benötigte die so entstehende „Gegenmacht“ auch die richtigen Argumente, so Günter Altner: Der hohe „Grad an Allgemeinheit“ erlaube es, „[d]ie vom Bürger geäußerten Befürchtungen […] als emotionale Einrede schnell abzutun“, sodass sie „keinen politischen Regelungsbedarf“, „kein präventives Verbot oder welche Maßnahmen auch immer“ rechtfertigen würden (Altner 1986: 1). Um die nicht-wissenschaftlichen Akteur*innen mit den „richtigen“ Entgegnungen und Argumentationsstrategien auszustatten, war das Symposion neben seiner inhaltlichen Dimension auch als Aufführung wissenschaftlicher Praxis inszeniert: Auf der Bühne kamen Wissenschaftler*innen, Expert*innen und Spezialist*innen zusammen, um vor einem Publikum Fakten zu präsentieren, Argumente und Gegenargumente darzulegen; sie entkräfteten, reflektierten, prüften, wogen ab, hinterfragten, erörterten, diskutierten, stimmten zu, äußerten Widerspruch oder begründeten Dissens. Die Vorträge wurden zudem durch wissenschaftliche Praktiken zur Erzeugung von epistemischer Gewissheit, wie Zeichnungen, Tabellen, Experimentieren usw. bereichert (Schmidt 2019: 559). Dem nicht-wissenschaftlichen Publikum kam dabei ebenfalls eine tragende Rolle zu: Auch wenn die Anwesenden natürlich über eine gewisse Vorbildung verfügten, repräsentierten ihre Fragen und Kommentare die Sorgen, Ängste und Erwartungen der Bevölkerung, zu denen die Referent*innen dann noch einmal pointiert Stellung beziehen konnten. So vermittelten sie einerseits inhaltlich wissenschaftliche Argumente gegen die Gentechnologie und demonstrierten dem Publikum gleichzeitig die Praxis wissenschaftlich-kritischer Methoden, was sich schon anhand der Beweisführung und Argumentation innerhalb der Vorträge, umso mehr aber in den Positionierungen und Gegenargumenten der Podiumsdiskussionen zeigte.^32^ Das Symposion reproduzierte damit einerseits wissenschaftliche Deutungshoheit in Bezug auf technologie- und wissenschaftspolitische Fragen, war aber andererseits auch Ort, an dem Wissenschaft und deren Deutungshoheit öffentlich verhandelt wurden. Die Struktur der Tagung, beginnend bei naturwissenschaftlichen Beobachtungen und endend mit den Panels zu den Möglichkeiten rechtlicher und politischer Rahmung der Gentechnologie, spiegelt wider, dass die Veranstalter*innen die politische Entscheidung den zuvor bereitgestellten naturwissenschaftlichen Erkenntnissen nachordneten. Diese Struktur findet sich auch in den Vorträgen zu Sicherheitsaspekten der Gentechnologie wieder, die von der Beobachtung biologischer Vorgänge ausgehend politischen Handlungsbedarf ableiteten.^33^ Die politischen Entscheidungen sind den naturwissenschaftlichen Erkenntnissen nachgeordnet. Zudem kommt den in der Forschung arbeitenden Wissenschaftler*innen bei Bartels die besondere Rolle zu, allgemeine Handlungsnotwendigkeiten aufzuzeigen, politischen Entscheidungsbedarf zu formulieren und den entscheidenden Instanzen Daten und Argumente zur Meinungsbildung zur Verfügung zu stellen (Bartels 1986: 79–83; Kollek et al. 1986a: 84–88). Das Ziel der am Symposion teilnehmenden Wissenschaftler*innen war eben auch, die eigene Deutungshoheit in Bezug auf die Bewertung von Technologie beziehungsweise von technologiepolitischen Entscheidungen, die zunehmend in Frage gestellt wurde, sicherzustellen und auszuweiten. Auch auf dem Symposion zeigten sich Divergenzen zwischen den sich mehr im Feld der Politik und den sich mehr in den Wissenschaften verortenden Akteur*innen. Zum Beispiel ging Erika Hickel nicht davon aus, dass sich politischer Handlungsdruck nur über wissenschaftliche Argumente generieren ließe. Nach eigenen Angaben war sie „enttäuscht und entsetzt“, dass auf dem Symposion – wie zum Beispiel von Regine Kollek – Risiko- und Sicherheitsforschung zur Grundlage der Technikbewertung gemacht wurden (Kollek et al. 1986a: 228). Sie forderte „ein dezidiertes ‚Nein‘“ zur Gentechnologie „[n]icht weil die Wissenschaftler Gefahren aufzeigen, nicht weil es irgendwelche Nebenwirkungen gibt, sondern weil wir diese Art von Umgang mit der Natur nicht wollen.“ (Kollek et al. 1986a: 228). Im Gegensatz zu Altner und anderen Tagungsteilnehmer*innen, die wissenschaftliche Argumente „öffentlich formulieren und politisch zum Tragen bringen“ (Bartels 1986: 85) wollten, trat Hickel dafür ein, „außertechnische und außerwissenschaftliche Wertvorstellungen – Wertentscheidungen massiv in die Technikdiskussion einzubringen“ (Kollek et al. 1986a: 228). Gerade Laien, wie zum Beispiel die „diversen Frauengruppen im Land“, lieferten laut Hickel die „qualifiziertesten, engagiertesten und unorthodoxesten Problemlösungsversuche“ (Kollek et al. 1986a: 228).^34^ Das Symposion lässt sich somit auch als Reaktion auf die zeitgenössische wissenschaftskritische Haltung verstehen und gleichzeitig als Versuch, die wissenschaftliche Deutungsmacht in Bezug auf technologiepolitische Entscheidungen zu erhalten.

In Abgrenzung zu den nicht-öffentlich stattfindenden Anhörungen der Enquete-Kommission wurde das Symposion explizit als öffentlich konzipiert. Die Frage der öffentlichen Beteiligung an der Kommissionsarbeit war zu deren Beginn diskutiert und negativ entschieden worden. Die Grünen kritisierten dieses Vorgehen aufgrund des Mangels an Transparenz sowie der fehlenden gesellschaftlichen Einbeziehung (Vowe 1991: 661–663). Angesichts der konsensorientierten, gegensätzliche Positionen einebnenden Kommissionsarbeit wollten die Veranstalter*innen des Heidelberger Symposions öffentlich auf die möglichen Gefahrenpotentiale der Gentechnologie hinweisen. Dabei ging es ihnen um die außenwirksame Darstellung wissenschaftlicher Erkenntnisse mitsamt ihren argumentativen Auseinandersetzungen und nicht etwa um die interne Erarbeitung schneller und innovativer Lösungen für drängende Fragen, wie sie Margaret Mead als Ziel der *small conference* beschrieb. Das Symposion stellte angesichts der intern ablaufenden und über Jahre andauernden Lösungsfindung durch die Enquete-Kommission ein wirksames Format dar, um kritische Positionen zur Gentechnologie und ungeklärte Fragen zu gentechnischen Anwendungen schon vor Veröffentlichung des Kommissionsberichts in die Öffentlichkeit zu tragen. Daran konnten auch Personen – aktiv durch den Besuch des Symposions oder passiv über den sehr zeitnah publizierten Tagungsband – teilhaben, die nicht zum erlesenen Kreis der Kommissionsmitglieder gehörten. Darüber hinaus wollten die Veranstalter*innen in Anbetracht der in der Kommission nicht so wie erwartet verlaufenden Diskussionen, schon einmal den Boden für eine kritische Aufnahme des Abschlussberichts nähren, um so ein Gewicht in Bezug auf die folgenden politischen Entscheidungen zu gewinnen. Regine Kollek war zum Beispiel, im Gegensatz zur Mehrzahl der Kommissionsmitglieder, der Ansicht, dass Freisetzungen gentechnologisch veränderter Organismen prinzipiell nicht beherrschbar seien und bewertete die mit der Gentechnologie einhergehenden Risiken weitaus höher (Vowe 1991: 615). Sie musste deshalb davon ausgehen, dass ihre Position im Abschlussbericht nicht ausreichend repräsentiert würde. In ihrer Funktion im wissenschaftlichen Sekretariat der Enquete-Kommission konnte sie ihre Ansichten nur indirekt in die Diskussionen einbringen. Sie überschritt somit ihre Rolle als wissenschaftliche Expertin und Beraterin des Bundestages, indem sie außerhalb ihrer Arbeit der Enquete-Kommission ihre Ansichten politisch und öffentlich zum Tragen brachte, was nicht von allen Kommissionsmitgliedern wohlwollend aufgenommen wurde (Vowe 1991: 662). Wie bereits erläutert, waren insbesondere die Grünen auf die öffentlichkeitswirksame Inszenierung ihrer Positionen angewiesen, auch weil sie mit nur einem Mitglied in der Kommission schwach vertreten waren. Dem Ansinnen, ihren wissenschaftlichen Stab zu den Sitzungen zuzulassen und diesem ein Anwesenheits- und Rederecht zuzusprechen, wurde seitens der Enquete-Kommission nicht entsprochen (Vowe 1991: 604).^35^ Die Grünen waren einerseits von der nicht öffentlichen und technikorientierten Arbeitsweise der Kommission enttäuscht, die zum Beispiel ein Nachdenken über etwaige Alternativen zu gentechnischen Eingriffen gänzlich ausschloss (Die Grünen 1987).

Über die öffentliche Veranstaltung des Symposions wollten die Veranstalter*innen auch anderen Forscher*innen demonstrieren, dass eine kritische wissenschaftliche Auseinandersetzung zur Gentechnologie bereits existierte, an der man sich auch als Mitglied des institutionalisierten Wissenschaftssystems beteiligen konnte. In ihren Augen war es angesichts der strukturellen Bedingungen insbesondere für junge Forscher*innen fast unmöglich, sich kritisch zu äußern, ohne vom Wissenschaftsbetrieb ausgestoßen zu werden. Auf der Tagung wurde die längst in Planung befindliche Gründung eines Netzwerks vorgeschlagen, das den in den Laboren tätigen Wissenschaftler*innen die Möglichkeit geben sollte, ihre Einwände in einem geschützten Raum zu kommunizieren und mit anderen Forscher*innen zu diskutieren (Kollek 1986: 232). Nach amerikanischem Vorbild wollte man auch in Deutschland eine Plattform gründen, über die sich kritische Wissenschaftler*innen vernetzten konnten, um sich aus den bestehenden finanziellen Abhängigkeitsverhältnissen in der Forschung zu emanzipieren. Im Sommer des Jahres 1986 gründete sich das nach dem bereits erwähnten Gentechnik-kritischen Publikationsorgan (GID) benannte Gen-ethische Netzwerk e. V., in dem sich eine Vielzahl der Tagungsbeteiligten aus den unterschiedlichen gesellschaftlichen Funktionsbereichen, unter anderem Günter Altner, Regine Kollek, Beatrix Tappeser, Rainer Hohlfeld, Erika Hickel, Arnim von Gleich und viele mehr vernetzten. Zum Leidwesen der Gründer*innen beteiligten sich langfristig kaum Mitglieder aus staatlich oder industriell finanzierten Forschungseinrichtungen innerhalb des Netzwerks. Gerade aber für die Mitglieder der Grünen bot es die Chance, sich unabhängig von der offiziellen Parteilinie mit der Gentechnik auseinanderzusetzen.^36^

## Fazit

Wie reagierten die am Heidelberger Symposion beteiligten Wissenschaftler*innen angesichts der Herausforderungen neuer technologischer Entwicklungen auf die gestiegenen Erwartungen an die Wissenschaft, beziehungsweise wie trugen sie selber zum Phänomen einer umstrittener werdenden Expertise bei? Diese eingangs gestellten Fragen möchte ich nun aufgreifen, um das hier vorgestellte Fallbeispiel noch einmal im größeren Kontext der Wissensgeschichte zu verorten. In dem Beitrag ist deutlich geworden, dass das Heidelberger Symposion nicht nur als öffentlich wirksamer Vollzug einer wissenschaftlich-epistemischen Praxis, sondern auch als politisches Aktivwerden der beteiligten Wissenschaftler*innen betrachtet werden kann. Neben der öffentlichen Meinungsbildung in Bezug auf die Gentechnologie, die die Organisator*innen als Ziel der Veranstaltung definierten, ging es auf dem Symposion auch darum, einerseits wissenschaftliche Deutungshoheit gegenüber einer kritischen Öffentlichkeit zu sichern sowie andererseits die eigenen wissenschaftlichen Deutungen gegenüber Gentechnik-Befürworter*innen aus der Politik und der durch Staat und Industrie geförderten Wissenschaft politisch durchzusetzen. Die Mobilisierung der Öffentlichkeit, deren „Informationsbedürfnis“ die Organisator*innen mit der Veranstaltung ganz gezielt zu wecken suchten, stellte ein wichtiges Instrument dar, um den eigenen Positionen politisches Gewicht zu verleihen. Das Symposion verortete sich dementsprechend im Kontext der gesellschaftlichen Auseinandersetzungen um die Rolle und Deutungshoheit der Wissenschaften in politischen Fragen. Gerade die Abgrenzung zur staatlich und industriell institutionalisierten Forschung und der Verweis auf die eigene wissenschaftliche Unabhängigkeit wurden zu einem politischen Argument. Gleichzeitig borgten sich die Veranstalter*innen aber auch Autorität aus dem institutionalisierten Wissenschaftssystem, um ihre eigene Position zu untermauern und dem Vorwurf der Emotionalität, der seitens der institutionalisierten Forschung vorgebracht wurde, zu entgehen. Die Referent*innen waren an führenden (universitären) Forschungseinrichtungen tätig und besaßen internationales wissenschaftliches Renommee. Kollek, Tappeser und Altner hatten akademische Ausbildungen erfolgreich absolviert und auch wenn sie zum Zeitpunkt des Symposions nicht an staatlich finanzierten Forschungseinrichtungen arbeiteten, so verfügten sie dennoch über wissenschaftliche Reputation. Wie die Analyse zeigte, wandten sie sich auch nicht ab von wissenschaftlichen Methoden oder Bewertungsmaßstäben, sondern reproduzierten sie. Dies zeigt sich besonders am Beispiel Regine Kollek, die 1995 zur ersten Professorin für Technologiefolgenabschätzung der modernen Biotechnologie in der Medizin an die Universität Hamburg berufen wurde.

Die öffentliche Etablierung alternativer wissenschaftlicher Perspektiven und Strukturen trug unmittelbar zur Erhöhung des Rechtfertigungsdrucks auf Wissenschaft und Forschung bei, wobei man bemüht war, die Pluralisierung wissenschaftlicher Deutungen nicht mit einem generellen Autoritätsverfall der Wissenschaften einhergehen zu lassen (Weingart 2001: 335–355).

## Footnotes

1 Vgl. Deutscher Bundestag (1987).

2 Auch in neueren wissens- und wissenschaftsgeschichtlichen Studien tauchen Expert*innen häufig nur in ihrer politikberatenden Funktion auf, vgl. Oppenheimer et al. (2019). Das Konzept der Gegenexpert*innen lässt sich hier nicht wirklich anwenden, vgl. dazu Güttler in Special Section: Gegenwissen. Vgl. Germann & Held & Wulz: Einleitung Scientific Political Activism.

3 Diese Definition von „Wissensgesellschaft“ ist keineswegs eindeutig. Neben dieser beispielsweise von Stehr (1994) vertretenen Definition, definieren andere Autor*innen „Wissensgesellschaft“ über den wachsenden Einfluss moderner Informationstechnologien für die Sammlung und Distribution von Wissen (Fried & Süßmann 2001).

4 Jüngst nachgewiesen am Beispiel der „Neuen Rechten“ (Stadler et al. 2021).

5 Hervorzuheben als zwei der wenigen spezifisch historischen Werke zur öffentlichen Debatte um die Gentechnologie in der Bundesrepublik Deutschland sind die etwa zeitgleich erschienenen und einschlägigen Arbeiten von Samia Salem (2013) und Thomas Wieland (2012).

6 Mit dem Begriff *scientific political activism* werden unterschiedliche gesellschaftspolitische Interventionen von Wissenschaftler*innen bezeichnet, die Wissenschaft als politische Strategie mobilisieren, vgl. Germann & Held & Wulz: Einleitung Special Section.

7 Zuvor wurde die Forschung mit rekombinanter, sprich künstlich hergestellter DNA bereits in kleineren innerwissenschaftlichen Kreisen hinsichtlich ihrer potentiellen Risiken diskutiert (Salem 2013: 63–64).

8 Durch architektonische Konzepte räumlicher Vernetzung zwischen staatlicher Forschung und den ortsansässigen Unternehmen sollte ein schneller Wissenstransfer zwischen Grundlagenforschung und industrieller Anwendung befördert werden, vgl. Gschaider (2018).

9 Auch aufgrund der starken Proteste konnte die Anlage erst 1990 im Zuge der Einführung des Gentechnikgesetzes bewilligt werden, vgl. Thielemann (1998).

10 Enquete-Kommissionen dienen dem allgemeinen Informationsbedürfnis des Parlaments. Sie ermöglichen eine regierungsunabhängige Wissenserhebung zu gesellschaftlich relevanten Fragen und sollen das Parlament in wichtigen Sachentscheidungen unterstützen, wodurch sie auch zur Versachlichung gesellschaftlicher Kontroversen beitragen, vgl. Altenhof (2002: 49–60).

11 Vgl. Vowe (1991: 577–684). Verwiesen sei an dieser Stelle auch auf das Dissertationsprojekt von Anna Klassen (Universität Jena), das die Aushandlung von Deutungshoheit über die Entwicklungen und Risiken der Biowissenschaften unter anderem anhand der Enquete-Kommission untersucht, URL: https://www.kooperation-und-konkurrenz.geschichte.uni-muenchen.de//teilprojekte1/phase1/tp-7/index.html (14.09.2022).

12 So erinnert sich zumindest der damalige Grünen-Politiker Benedikt Härlin in einem Gespräch vom 27.07.2020, geführt und dokumentiert von Anna Maria Schmidt (im Folgenden mit A.M.S. abgekürzt).

13 URL: https://www.biogum.uni-hamburg.de/personen/forschungsgruppe-medizin/kollek.html (31.08.2020).

14 Gespräch mit Beatrix Tappeser vom 20.08.2020, geführt und dokumentiert von A.M.S.

15 Gespräch mit Beatrix Tappeser, siehe Fußnote 14.

16 Gespräch mit Beatrix Tappeser, siehe Fußnote 14.

17 So erinnert sich Tappeser an die breite Wirksamkeit des Bandes, aufgrund dessen die geäußerten Bedenken von den politischen Verantwortlichen nicht mehr beiseitegeschoben werden konnten. Gespräch mit Beatrix Tappeser vom 20.08.2020, geführt und dokumentiert von A.M.S.

18 Gespräch mit Beatrix Tappeser vom 20.08.2020, geführt und dokumentiert von A.M.S.

19 Gespräch mit Beatrix Tappeser vom 20.08.2020, geführt und dokumentiert von A.M.S. Tappeser wies zudem darauf hin, dass die hehren Ideen der Asilomar-Konferenz letztendlich von Nutzungsinteressen vereinnahmt wurden, denen es gelang, die Debatte auf bestimmte Punkte und auch Kreise zu beschränken, vgl. Wieland (2012: 71–72).

20 Vgl. Stadler et al. (2020).

21 Die Altner-Stiftung (seit 2003 Altner-Combecher-Stiftung für Ökologie und Frieden) wurde 1982 von Günter Altner und seiner Frau gegründet und setzt sich seitdem für die Förderung von Wissenschaft und Forschung in Umweltfragen ein, vgl. Deutsches Stiftungszentrum. Bei der Deutschen Umweltstiftung handelt es sich um eine 1982 gegründete unabhängige Bürgerstiftung zur Unterstützung des praktischen Umweltschutzes sowie zur Förderung des Umweltbewusstseins. Die Stiftung Mittlere Technologie entstand 1975. Ihr Aufgabenschwerpunkt liegt in der Verringerung der Umweltbelastung und der Förderung natur- und menschengerechter Technologien. 1991 wurde sie in die Stiftung Ökologischer Landbau (SÖL) integriert.

22 Gespräch mit Beatrix Tappeser vom 20.08.2020, geführt und dokumentiert von A.M.S.

23 Zumindest kann sich Tappeser nicht daran erinnern. Gespräch mit Beatrix Tappeser vom 20.08.2020.

24 Gespräch mit Beatrix Tappeser vom 20.08.2020.

25 Die Vorträge werden nach üblichem Vorgehen bei Sammelbänden jeweils nach Autornamen zitiert. Aufgrund der fehlenden Autor*innenangaben in Bezug auf die Diskussionen, wird hier der gesamte Tagungsband (Kollek et al. 1986a) mit genauer Seitenangabe zitiert.

26 Die Diskutant*innen bleiben im Tagungsband leider teilweise anonym.

27 Gespräch mit Beatrix Tappeser vom 20.08.2020, geführt und dokumentiert von A.M.S.

28 Dieser 1985 gegründete unabhängige Nachrichtendienst zum Thema Gen- und Biotechnologien existiert unter geänderter Herausgeberschaft bis heute: https://www.gen-ethisches-netzwerk.de/publikationen/gid (05.08.2022).

29 Siehe hier zum Beispiel die Vorträge von Steven Rose, Susan Wright, Regine Kollek, Ditta Bartels sowie die Podiumsdiskussion über die Entstehungsgeschichte von AIDS.

30 Kollek et al. (1986a: 94).

31 So Hickel in der Abschlussdiskussion (Kollek et al. 1986a: 228).

32 Siehe exemplarisch: Kollek (1986: 49–69).

33 Exemplarisch Bartels, die ein Programm zur besseren Risikoabschätzung von experimentellen Arbeiten mit Onkogenen vorschlägt, das ausgehend von Datenerhebungen durch mit der nötigen Expertise ausgestattete Wissenschaftler*innen, „Maßnahmen zur Gewährleistung einer sicheren Arbeitsumwelt“ entwickeln soll.

34 Auch die amerikanische Aktivistin Linda Bullard, die sich als Koordinatorin der von Jeremy Rifkin gegründeten „Foundation on Economic Trends“ gegen die Gentechnologie engagierte, vertrat in Heidelberg die Position, dass die Beteiligung an der Debatte nicht von Disziplinenzugehörigkeiten abhängig gemacht werden dürfe (Bullard 1986: 36).

35 Die etwas marginalisierte Position der Grünen in der Enquete-Kommission führte zu einem Sondervotum, das vom wissenschaftlichen Stab, unter anderem von Beatrix Tappeser. Aufgrund der in dem Votum vertretenen Positionen erkennt Vowe zudem die Mitarbeit des kommissionseigenen wissenschaftlichen Sekretariats (Vowe 1991: 604).

36 Gespräch mit Benedikt Härlin vom 27.07.2020, geführt und dokumentiert von A.M.S.

## References

[CR1] Altenhof R (2002). Die Enquete-Kommissionen des deutschen Bundestages.

[CR2] Altner G, Kollek R, Tappeser B, Altner G (1986). Einführung in das öffentliche Fachsymposion: „Die ungeklärten Gefahrenpotentiale der Gentechnologie. Die ungeklärten Gefahrenpotentiale der Gentechnologie. Dokumentation eines öffentlichen Fachsymposions vom 7.–9. März 1986 in Heidelberg.

[CR3] Anonym 1981. Genetik: „Dutzendlinge, viele Dutzendlinge“. *Spiegel* (3): 153–154.

[CR4] Anonym 1986. Ökologiekongress in Heidelberg. *GID* 2 (14): 1.

[CR5] Barben D (2007). Politische Ökonomie der Biotechnologie. Innovation und gesellschaftlicher Wandel im internationalen Vergleich.

[CR6] Bartels D, Kollek R, Tappeser B, Altner G (1986). Genetische Manipulation von Krebsgenen. Die ungeklärten Gefahrenpotentiale der Gentechnologie. Dokumentation eines öffentlichen Fachsymposions vom 7.–9. März 1986 in Heidelberg.

[CR7] Beljan M (2015). AIDS-Geschichte als Gefühlsgeschichte. Aus Politik und Zeitgeschichte.

[CR8] Berg P, Baltimore D, Boyer HW, Cohen SN, Davis RW, Hogness DS, Nathans D, Roblin R, Watson JD, Weissman S, Zinder ND (1974). Potential biohazards of recombinant DNA molecules. Science.

[CR9] Berg P, Baltimore D, Brenner S, Roblin R, Singer MF (1975). Asilomar conference on recombinant DNA molecules. Science.

[CR12] de Boer J-H, de Boer J-H (2019). Praktiken, Praxen, Praxisformen, oder: Von Serienkillern, verrückten Wänden und der ungewissen Zukunft. Praxisformen. Zur kulturellen Logik von Zukunftshandeln.

[CR10] Brandt C (2010). Zeitschichten des Klons. Anmerkungen zu einer Begriffsgeschichte. Berichte der Wissenschaftsgeschichte.

[CR11] Bullard L, Kollek R, Tappeser B, Altner G (1986). Die öffentliche Auseinandersetzung um die Gentechnologie in den USA. Die ungeklärten Gefahrenpotentiale der Gentechnologie. Dokumentation eines öffentlichen Fachsymposions vom 7.–9. März 1986 in Heidelberg.

[CR13] Deutscher Bundestag 1987. *Bericht der Enquete-Kommission „Chancen und Risiken der Gentechnologie“*, 06.01.1987. URL: https://dip21.bundestag.de/dip21/btd/10/067/1006775.pdf (30.08.2020).

[CR15] Die Grünen im Bundestag (1987). Sondervotum zum Bericht der Enquete-Kommission „Chancen und Risiken der Gentechnologie“. Bericht der Enquete-Kommission „Chancen und Risiken der Gentechnologie“.

[CR16] Dolata U (1996). Politische Ökonomie der Gentechnik. Konzernstrategien, Forschungsprogram, Technologiewettläufe.

[CR17] Espahangizi K, Wulz M (2020). Introduction. The political and the epistemic in the twentieth century: historical perspectives. KNOW: A Journal on the Formation of Knowledge.

[CR18] Fried J, Süßmann J (2001). Revolutionen des Wissens. Von der Steinzeit zur Moderne.

[CR22] Geißler E, Anton A, Schetsche M, Miachel KW (2014). AIDS und seine Erreger – ein Gespinst von Hypothesen, Erkenntnissen und Verschwörungstheorien. Konspiration. Soziologie des Verschwörungsdenkens.

[CR19] Gill B, Roth R, Rucht D (2008). Kampagnen gegen Bio- und Gentechnologie. Die sozialen Bewegungen in Deutschland seit 1945. Ein Handbuch.

[CR20] Grießhammer R, Michelsen G, Simonis UE, de Witt S (2001). Forschung in der Quetschzone des Fortschritts. Zur Gründung und zum Erfolg des Öko-Instituts. Ein Grenzgänger der Wissenschaften. Aktiv für Natur und Mensch. Festschrift für Günter Altner zum 65. Geburtstag.

[CR21] Gschaider D, Bernhardt M, Blösel W, Brakensiek S, Scheller B (2018). Bauen für die Forschung der Zukunft. Raumstrategien gentechnologischer Industrieforschung in den 1980er Jahren. Möglichkeitshorizonte. Zur Pluralität von Zukunftserwartungen und Handlungsoptionen in der Geschichte.

[CR23] Hansen F, Kollek R (1985). Gen-Technologie. Die neue soziale Waffe.

[CR25] Hickel E (1985). Gefahren der Genmanipulation. Schlussfolgerungen aus der Arbeit in der Enquete-Kommission des Bundestages. Blätter für deutsche und internationale Politik.

[CR26] Idel A, Kamphausen R, Kollek R, Tappeser B, Altner G (1986). Angepasste Landrassen – oder Nutztiere aus der Retorte? – Der fragwürdige Beitrag der Gentechnologie zur Tierzüchtung. Die ungeklärten Gefahrenpotentiale der Gentechnologie. Dokumentation eines öffentlichen Fachsymposions vom 7.–9. März 1986 in Heidelberg.

[CR27] Illmensee K, Hoppe P (1981). Nuclear transplantation in mus musculus: developmental potential of nuclei from preimplantation embryos. Cell.

[CR29] Kampe D (1986). Wir können uns eine eigene Meinung leisten. Spiegel.

[CR31] Kollek R, Hansen F, Kollek R (1985). Die molekulare Definition des Menschen. Forschungsstand und Perspektiven. Gen-Technologie. Die neue soziale Waffe.

[CR32] Kollek R (1985). Zerschneide und herrsche. Von den neuen Reproduktionstechniken, den Visionen der Gentechniker und den Instrumenten der Biopolitik. Feministische Studien.

[CR33] Kollek R, Kollek R, Tappeser B, Altner G (1986). Sicherheitsaspekte der experimentellen Arbeit mit Retroviren. Die ungeklärten Gefahrenpotentiale der Gentechnologie. Dokumentation eines öffentlichen Fachsymposions vom 7.–9. März 1986 in Heidelberg.

[CR34] Kollek R, Tappeser B, Altner G (1986). Die ungeklärten Gefahrenpotentiale der Gentechnologie. Dokumentation eines öffentlichen Fachsymposions vom 7.–9. März 1986 in Heidelberg.

[CR35] Kollek R, Tappeser B, Altner G, Kollek R, Tappeser B, Altner G (1986). Vorwort. Die ungeklärten Gefahrenpotentiale der Gentechnologie. Dokumentation eines öffentlichen Fachsymposions vom 7.–9. März 1986 in Heidelberg.

[CR36] Maurer M (1986). Machbarkeit und Verantwortung. Gentechnologie in der Kritik. AUF – Eine Frauenzeitschrift.

[CR37] Mead M, Byers P (1968). The small conference. An innovation in communication.

[CR38] Öko-Institut 2017. 40 Jahre Öko-Institut – Highlights der Institutsgeschichte. URL: https://www.oeko.de/fileadmin/oekodoc/HG-Papier_40-Jahre-Oeko-Institut.pdf (01.09.2020).

[CR39] Oppenheimer M, Oreskes N, Jamieson D, Brysse K, O’Reilly J, Shindell M, Wazeck M (2019). Discerning Experts: The Practices of Scientific Assessment for Environmental Policy.

[CR40] Riesenhuber H, Der Bundesminister für Forschung und Technologie (1984). Wortprotokoll des Fachgesprächs über ethische und rechtliche Anwendung zellbiologischer und gentechnischer Methoden am Menschen am 14. und 15. September 1983 im Bundesministerium für Forschung und Technologie. Ethische und rechtliche Probleme der Anwendung zellbiologischer und gentechnischer Methoden am Menschen.

[CR41] Roose J (2002). Made by Öko-Institut: Wissenschaft in einer bewegten Umwelt.

[CR42] Rose S, Kollek R, Tappeser B, Altner G (1986). Gentechnologie und biologische Waffen. Die ungeklärten Gefahrenpotentiale der Gentechnologie. Dokumentation eines öffentlichen Fachsymposions vom 7.–9. März 1986 in Heidelberg.

[CR43] Rucht D (1988). Gegenöffentlichkeit und Gegenexperten. Zur Institutionalisierung des Widerspruchs in Politik und Recht. Zeitschrift für Rechtssoziologie.

[CR44] Salem S (2013). Die öffentliche Wahrnehmung der Gentechnik in der Bundesrepublik Deutschland seit den 1960er Jahren.

[CR45] Schmidt AM, de Boer J-H (2019). „Ich glaube, wir werden unser gentechnologisches Tschernobyl erleben.“ Das Katastrophenpotential der Gentechnik. Praxisformen. Zur kulturellen Logik von Zukunftshandeln.

[CR46] Schmidt AM (2020). Organisation von Widerstand. Der Kongress »Frauen gegen Gentechnik und Reproduktionstechnik« (Bonn, 19.–21.04.1985). Ariadne.

[CR47] Selvage D, Nehring C (2014). Die AIDS-Verschwörung. Das Ministerium für Staatssicherheit und die AIDS-Desinformationskampagne des KGB.

[CR48] Singer M, Söll D (1973). Guidelines for DNA Hybrid Molecules. Science.

[CR49] Stadler, Max, Nils Güttler, Niki Rhyner, Mathias Grote, Fabian Grütter, Tobias Scheidegger, Martina Schlünder, Anna Maria Schmidt, Susanne Schmidt, Alexander von Schwerin, Monika Wulz und Nadine Zberg 2020. Gegeninstitute. In: *Gegen|Wissen* (Cache 01). URL: https://cache.ch/gegenwissen/maschinensturm/protest/gegeninstitute (31.08.2020).

[CR50] Stadler M, Steuwer J, Wulz M, Stadler M, Steuwer J, Wulz M (2021). „Rechtes Wissen“ – Eine Problematisierung. Æther 06: Rechtes Wissen: Konstellationen zwischen Universität und Politik.

[CR51] Stehr N (1994). Arbeit, Eigentum und Wissen: zur Theorie von Wissensgesellschaften.

[CR52] Steindor M, Emmrich M (1999). Kritik als Programm. 15 Jahre grüne Gentechnologiepolitik im Deutschen Bundestag. Im Zeitalter der Bio-Macht. 25 Jahre Gentechnik – eine kritische Bilanz.

[CR53] Tappeser B, Die Grünen im Bundestag, AK Frauenpolitik & Sozialwissenschaftliche Forschung und Praxis für Frauen e. V. (1986). Auf dem Weg zum industriellen Normmenschen?. Frauen gegen Gentechnik und Reproduktionstechnik. Dokumentation zum Kongreß vom 19.–21.04.1985 in Bonn.

[CR54] Thielemann H, Renn O, Hampel J (1998). Kommunikation im Konflikt um die gentechnische Insulinherstellung bei der Hoechst AG. Kommunikation im Konflikt. Fallbeispiele aus der Chemie.

[CR55] Vowe G (1989). Die Sicherung öffentlicher Akzeptanz. Verlauf, Struktur und Funktion der Enquete-Kommission „Chancen und Risiken der Gentechnologie“. Politische Bildung.

[CR56] Vowe, Gerhard 1991. *Technik im parlamentarischen Diskurs. Die Enquete-Kommissionen des Deutschen Bundestages zum Verhältnis von Technik und Politik*. URL: https://www.phil-fak.uni-duesseldorf.de/fileadmin/Redaktion/Institute/Sozialwissenschaften/Kommunikations-_und_Medienwissenschaft/Vowe/diskurs-1.pdf und https://www.phil-fak.uni-duesseldorf.de/fileadmin/Redaktion/Institute/Sozialwissenschaften/Kommunikations-_und_Medienwissenschaft/Vowe/diskurs_anhaenge.pdf (14.09.2022).

[CR57] Weingart P (2001). Die Stunde der Wahrheit. Zum Verhältnis der Wissenschaft zu Politik, Wirtschaft und Medien in der Wissensgesellschaft.

[CR58] Wieland T (2009). Neue Technik auf alten Pfaden? Forschungs- und Technologiepolitik in der Bonner Republik. Eine Studie zur Pfadabhängigkeit des technischen Fortschritts.

[CR59] Wieland T, Weitze M-D, Pühler A, Heckl WM, Müller-Röber B, Renn O, Weingart P, Wess G (2012). Rote Gentechnik und Öffentlichkeit. Von der grundlegenden Skepsis zur differenzierten Akzeptanz. Biotechnologie-Kommunikation. Kontroversen, Analysen, Aktivitäten.

[CR60] Wright S, Kollek R, Tappeser B, Altner G (1986). Die Sozialgeschichte der Kontroverse um die rekombinante DNS in den USA. Die ungeklärten Gefahrenpotentiale der Gentechnologie. Dokumentation eines öffentlichen Fachsymposions vom 7.–9. März 1986 in Heidelberg.

